# Melatonin-Activated Receptor Signaling Pathways Mediate Protective Effects on Surfactant-Induced Increase in Jejunal Mucosal Permeability in Rats

**DOI:** 10.3390/ijms221910762

**Published:** 2021-10-05

**Authors:** Karsten Peters, David Dahlgren, Hans Lennernäs, Markus Sjöblom

**Affiliations:** 1 Department of Neuroscience, Gastrointestinal Physiology, Uppsala University, 751 24 Uppsala, Sweden; karsten.peters@farmbio.uu.se; 2 Department of Pharmaceutical Biosciences, Translational Drug Discovery and Development, Uppsala University, 752 37 Uppsala, Sweden; david.dahlgren@farmbio.uu.se (D.D.); hans.lennernas@farmbio.uu.se (H.L.)

**Keywords:** intestinal barrier dysfunction, single-pass intestinal perfusion, intestinal permeability, melatonin, luzindole, mecamylamine, gastrointestinal physiology

## Abstract

A well-functional intestinal mucosal barrier can be compromised as a result of various diseases, chemotherapy, radiation, and chemical exposures including surfactants. Currently, there are no approved drugs targeting a dysfunctional intestinal barrier, which emphasizes a significant medical need. One candidate drug reported to regulate intestinal mucosal permeability is melatonin. However, it is still unclear if its effect is primarily receptor mediated or antioxidative, and if it is associated with enteric neural pathways. The aim of this rat intestinal perfusion study was to investigate the mechanisms of melatonin and nicotinic acetylcholine receptors on the increase in intestinal mucosal clearance of ^51^Cr-labeled ethylenediaminetetraacetate induced by 15 min luminal exposure to the anionic surfactant, sodium dodecyl sulfate. Our results show that melatonin abolished the surfactant-induced increase in intestinal permeability and that this effect was inhibited by luzindole, a melatonin receptor antagonist. In addition, mecamylamine, an antagonist of nicotinic acetylcholine receptors, reduced the surfactant-induced increase in mucosal permeability, using a signaling pathway not influenced by melatonin receptor activation. In conclusion, our results support melatonin as a potentially potent candidate for the oral treatment of a compromised intestinal mucosal barrier, and that its protective effect is primarily receptor-mediated.

## 1. Introduction

The intestinal mucosa is a selective and dynamic barrier separating the luminal contents and the systemic circulation [1]. This barrier allows the transport of water, nutrients and ions while restricting the passage of harmful substances such as allergens, microbiota and toxins. From the apical to the basolateral side, the intestinal mucosal barrier consists of a mucus layer, a single layer of intestinal epithelial cells, and the underlying immune system. The intestinal epithelial cells primarily constitute enterocytes, which are involved in carrier-mediated electrolyte and nutrient transport in both the absorptive and secretory direction in order to uphold physiological functions in digestion and absorption. In addition, molecular paracellular diffusion across the epithelium is strictly regulated to uphold intestinal as well as systemic homeostasis [2,3]. The key components in this regulation are the tight junction proteins at the apical portion of the intestinal epithelial cells, which determine the epithelial transfer of hydrophilic molecules and electrolytes with low cell membrane permeability. These junction proteins are linked to the intracellular actin cytoskeleton, and thereby undergo continuous modification in response to luminal, hormonal and neural stimuli in order to regulate transmembrane solute transport [4,5].

A dysfunctional intestinal barrier is associated with a range of gastrointestinal (GI) and systemic diseases and disorders, such as inflammatory bowel disease, irritable bowel syndrome, type 1 diabetes, non-alcoholic fatty liver disease as well as anti-cancer treatment with chemotherapeutics, tyrosine kinase inhibitors and radiation [6]. Common observations with these conditions are intestinal inflammation, altered epithelial secretory and absorptive functions, as well as an increased mucosal permeability that may enable infiltration of harmful substances and bacterial translocation [7,8,9]. Increased mucosal permeability may also trigger the development and proliferation of many serious conditions including multi-organ failure [10], while other studies suggest that an increased intestinal permeability may contribute to further progression of leaky gut syndrome, but the mechanisms for this have not been fully described [11]. Nonetheless, reversing pathological increases in intestinal mucosal permeability may aid in combatting underlying GI diseases. It may also alleviate symptoms such as diarrhea, which is commonly observed in association with for instance irritable bowel syndrome and chemotherapy [12,13].

Melatonin released from the pineal gland has a significant physiological role in regulating the circadian rhythm of the body together with cortisol. When the melatonin concentration rises at night, the cortisol concentration is reduced to its trough values, and when the cortisol concentration reaches its peak value in the early morning, the melatonin concentration drops to its lowest. It is well known that abnormal concentration–time profiles of melatonin and cortisol rhythm results in problems with sleep [14]. Interestingly, the GI tract is the greatest source of extra-pineal melatonin, where it is synthesized by the enterochromaffin cells [15]. Melatonin has been shown to be a potent scavenger of free radicals, both through direct detoxification of reactive oxygen and nitrogen species as well as by stimulating antioxidant enzymes and suppressing the pro-oxidant enzymes [16]. In addition, its actions in the intestine is also mediated through binding to melatonin membrane G protein-coupled receptors. Two types of melatonin receptors, MT_1_ and the MT_2_, are expressed in the GI tracts of both rats and humans [17,18].

Melatonin increases duodenal mucosal bicarbonate secretion, reduces basal jejunal permeability, and is considered an important mediator of acid-induced secretion [19,20,21]. These melatonin effects are primarily receptor mediated rather than anti-oxidative, as they can be reduced by the administration of the melatonin receptor antagonist, luzindole [21]. As a GI protective drug, melatonin reduces ethanol- and radiation-induced increases of intestinal permeability. For ethanol, the melatonin effect is primarily receptor mediated, whereas for radiation it is mainly antioxidative [19,20].

Recent research from our group has also shown that melatonin reduces sodium dodecyl sulfate (SDS)-induced changes to the mucosal barrier in rats [22]. SDS is an anionic surfactant commonly used as a pharmaceutical excipient in many oral dosage forms. At high amounts in the intestinal lumen, SDS has the potential to alter epithelial barrier integrity and it has been shown to increase permeability in both the absorptive and secretive directions [23,24]. However, it is currently unclear if the capacity of melatonin to inhibit the surfactant-induced increase in intestinal permeability is receptor mediated or antioxidative, which calls for further investigations of the mechanisms involved [24].

Excitatory neurotransmission in the enteric nervous system is primarily mediated by nicotinic acetylcholine receptors [25]. These receptors play an important role in the physiological regulation of intestinal functions, such as duodenal motility, transmucosal fluid and electrolyte flux [26]. The receptors also mediate increases in mucosal permeability induced by luminal deoxycholic acid [27], and their blocking using hexamethonium completely abolishes the protective effect of melatonin on ethanol- and wine-induced increases in intestinal permeability [19]. In addition, it seems that α7 nicotinic acetylcholine receptors are involved in vagal influence on mucosal tight junctional ultrastructure [28]. Altogether, this makes these receptors a target of interest when studying intestinal mucosal permeability.

The main objective of this rat single-pass intestinal perfusion (SPIP) study was to investigate the role of melatonin receptors and nicotinic acetylcholine receptors on an increase in intestinal permeability induced by 15 min luminal exposure to SDS. The changes in jejunal epithelial permeability were evaluated by monitoring intestinal flux of ^51^Cr-labeled ethylenediaminetetraacetate (^51^Cr-EDTA), a well-established marker for studies of mucosal barrier integrity [29].

## 2. Results

Mean arterial blood pressure (MABP), and body temperature (37.5 ± 0.5 °C), remained stable and at a physiologically normal level in all groups. In the group treated with mecamylamine and SDS, the MABP decreased from 95 ± 3 mmHg (0–15 min) to 77 ± 7 mmHg (105–120 min) (Table 1).

**Figure 1:**
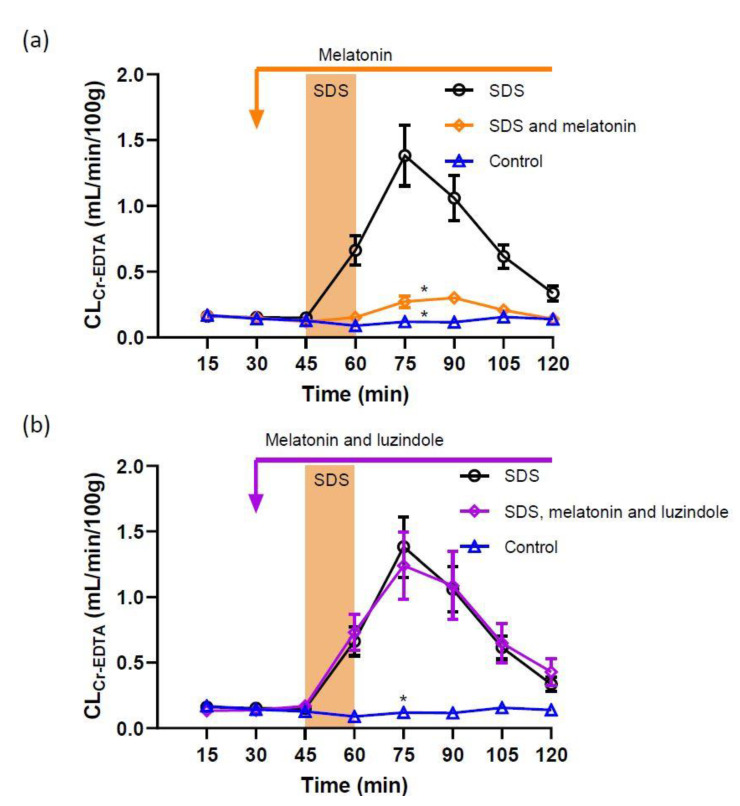
Effect of 5 mg/mL SDS in the luminal perfusate, between 45 and 60 min, on jejunal paracellular permeability (blood-to-lumen ^51^Cr-EDTA clearance (CL_Cr-EDTA_)). (**a**) SDS induced a significant increase in permeability, an effect that was abolished by addition of luminal melatonin (100 µM). (**b**) Adding luzindole (melatonin receptor antagonist) to the luminal perfusate at 100 µM completely inhibited the protective effect of melatonin (100 µM). Values are means (±SEM). * significantly (*p* < 0.05) lower response compared with the SDS group.

In the control group, i.e., animals perfused luminally with an isotonic phosphate-buffered solution, jejunal epithelial permeability was stable throughout the 120 min experiment (Figure 1a). The total CL_Cr-EDTA_ in the control group was 9.2 ± 1.3 mL/100 g. Perfusing the jejunal segment with an isotonic solution containing 5 mg/mL SDS for 15 min increased the total CL_Cr-EDTA_ from 9.2 ± 1.3 to 59.5 ± 7.7 mL/100 g (*p <* 0.01, Figure 1a). The addition of melatonin at a concentration of 100 µM to the luminal perfusate before, during and after SDS exposure significantly reduced (*p <* 0.01) the SDS-induced increase in total CL_Cr-EDTA_ when compared to SDS alone (from 59.5 ± 7.7 to 15.9 ± 1.8 mL/100 g, Figure 1a).

In order to assess the involvement of melatonin receptors on jejunal permeability, the melatonin receptor antagonist luzindole, was perfused luminally at 100 µM. Luzindole alone did not affect the basal permeability (total CL_Cr-EDTA_ of 10.3 ± 2.1 mL/100 g, not shown). However, luzindole perfused before, during and after SDS exposure abolished the inhibitory effect of luminal melatonin (100 µM) on the SDS-induced increase in paracellular permeability. In this group of animals, the total CL_Cr-EDTA_ was 60.1 ± 11.0 mL/100 g (Figure 1b) compared to 15.9 ± 1.8 mL/100 g in the group treated with melatonin (*p <* 0.05) (Figure 1a). This was an increase similar to animals exposed to SDS alone (*p >* 0.99, see Figure 1a,b).

In animals treated with luzindole before, during and after SDS exposure total CL_Cr-EDTA_ increased significantly compared to the control (from 9.2 ± 1.3 to 35.9 ± 8.8 mL/100 g, *p* < 0.05), an increase not significantly different from SDS alone (*p* > 0.05).

**Figure 2:**
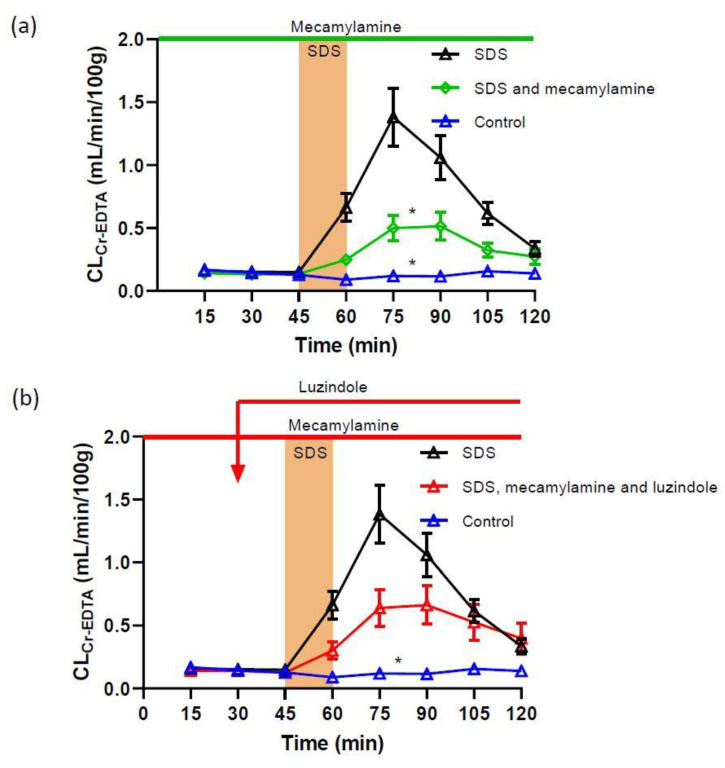
Effect of 5 mg/mL SDS in the luminal perfusate, between 45 and 60 min, on jejunal paracellular permeability (blood-to-lumen ^51^Cr-EDTA clearance (CL_Cr-EDTA_)). (**a**) Adding the non-selective, non-competitive nicotinic acetylcholine receptor antagonist mecamylamine to the luminal perfusate at 100 µM strongly reduced the SDS-induced increase in permeability. (**b**) Adding luzindole (melatonin receptor antagonist) at 100 µM did not alter the effect of mecamylamine. Values are means (±SEM). * significantly (*p* < 0.05) lower response compared with the SDS group.

To assess the involvement of nicotinic acetylcholine receptors in the SDS-induced increase in mucosal permeability, mecamylamine (nicotinic acetylcholine receptor antagonist) was perfused luminally at 100 µM before, during and after SDS exposure. The addition of mecamylamine did not affect the basal permeability in the extra 30 min control period (not shown). However, mecamylamine in the luminal perfusate reduced the SDS-induced increase in total CL_Cr-EDTA_ from 59.5 ± 7.7 to 26.8 ± 4.4 mL/100 g (Figure 2a), an increase significantly smaller compared to the response induced by SDS without mecamylamine treatment (*p <* 0.05). Adding luzindole to the luminal perfusate (100 µM) did not alter (*p* > 0.05), the effect of mecamylamine in response to luminal SDS, and resulted in a total CL_Cr-EDTA_ of 35.8 ± 4.4 mL/100 g (Figure 2b).

## 3. Discussion

Surfactants, such as SDS, have been investigated for their use as intestinal permeability enhancers to improve GI absorption of low permeability drugs [30]. The amphiphilic, surface-active properties of surfactants allow some of them to be incorporated into the cell membrane lipid bilayer, thus causing an increase in its fluidity [31]. In the case of SDS, increased luminal exposure may result in lysis of the cell membrane [32]. Combined, the membrane effects lead to a loss of membrane integrity and an increase in intestinal permeability of hydrophilic molecules and some polar drugs [33]. The effect of SDS on intestinal solute permeability is both concentration- and time-dependent and shows the biochemical and histological characteristics of intestinal mucosal injury [34,35]. Combined, this makes SDS a viable agent for studying dysfunction in the intestinal barrier.

The primary scope of the present study was to elucidate the mechanisms of melatonin in response to an acute increase in jejunal mucosal permeability induced by 15 min SDS exposure of the jejunum in a rat single-pass intestinal perfusion (SPIP) model [19,24]. It was clearly shown that melatonin attenuated the SDS-induced increase in jejunal mucosal permeability. This effect is previously reported [24], but it is unclear if it is mediated by melatonin receptors or by its antioxidative effects. To investigate the mechanisms we used luzindole, a potent melatonin receptor antagonist [36]. Luminal luzindole completely abolished the protective effect of melatonin, strongly suggesting that the acute effect of melatonin on the SDS-induced increase in membrane permeability is receptor mediated rather than antioxidative [17]. The mechanisms by which melatonin receptor activation protects against luminal surfactants thus seems to be similar to some of its physiological effects in the intestines. For instance, luzindole inhibits the effect of melatonin on the regulation of basal duodenal permeability, as well as its ability to increase bicarbonate secretion [21,37] and intestinal motility [38].

Many intestinal functions are controlled by nicotinic acetylcholine receptors in the enteric nervous system, such as duodenal motility and transmucosal fluid and electrolyte flux [26]. It is also reported that nicotinic receptor blockage reduces a hypotonicity-induced increase in duodenal mucosal permeability [39]. This mechanism may be related to the one in our study, where mecamylamine (a non-selective, non-competitive antagonist of nicotinic acetylcholine receptors) had a protective effect on an SDS-induced increase in permeability [40]. This is in contrast to permeability increases induced by ethanol and acid, where nicotinic receptor inhibition had no effect [19,41]. While the blockage of nicotinic receptors inhibits the effects of melatonin on ethanol-induced increases in duodenal permeability, basal duodenal permeability and mucosal bicarbonate transport [19,21], we showed that the effect of mecamylamine on SDS was not influenced by melatonin receptor inactivation, as luzindole did not modify this response. These differences may be due to the different modes of induction of permeability increases. These data suggest that the increase in mucosal permeability in response to SDS and hypotonicity is mediated by different mechanisms than for ethanol and acid, explaining differences in attenuation of the induced increases. For SDS and hypotonicity, they appear to be under physiological regulation and using similar neural pathways. Further elucidation of these pathways is under investigation.

The SPIP model has been extensively applied to investigate intestinal physiology and pathophysiology. It provides an intact morphology, blood supply, as well as neuro-endocrine and hormonal signaling, which is its main advantage compared to in vitro or ex vivo models. This ensures that there are limited effects on normal GI functions while physiological feedback processes necessary for maintaining a functional mucosal barrier are preserved. For instance, when subjected to hypotonic (20–200 mOsm) solutions, the rat duodenum and jejunum in the SPIP model show normal physiological responses [39,42,43]. The SPIP model has also been used to study the intestinal damage caused by acetazolamide and its influence on the permeability for model compounds [44] as well as the influence of the cholera toxin on the passive transport of small hydrophilic molecules in the lumen [45]. In addition, the GI tract of rats has been shown to be of good translational value for predicting intestinal drug absorption in humans [46,47]. Combined, this makes the SPIP model physiologically relevant for the study of intestinal barrier dysfunction and the development of novel treatment strategies.

Laparotomic surgery, as used in the rat SPIP model, compromises some normal intestinal functions. This state is called postoperative ileus, which is partly mediated by COX-2-derived intestinal prostacyclin. As such, administration of a selective COX-2 inhibitor can restore depressed intestinal functions, such as duodenal mucosal bicarbonate transport, permeability, motility, osmoregulation, and water transport [24,48,49,50]. In the present study, the selective COX-2 inhibitor parecoxib was used to enable investigations of physiological regulation of intestinal permeability. Additionally, the arterial blood pressure was continuously monitored in all animals in order to secure the viability of the anesthetized animals and to detect systemic effects of the study drugs. All animals in the present study had a systemic blood pressure within the physiological range. As expected, in rats that were administered mecamylamine, the systemic blood pressure was slightly lower than in control. However, the systemic effects were lower compared to similar drugs with a lower membrane permeability resulting in the need for systemic rather than local administration, such as hexamethonium.

Ahead, our current SPIP model for investigating the effects of drugs on intestinal mucosal permeability will be used to evaluate new treatments for various GI diseases and conditions. One example that currently lacks any effective treatment is chemotherapy-induced mucositis, a major side effect and safety issue associated with this anti-cancer therapy [51]. Its pathology is triggered by epithelial stem cell death, which results in a range of effects, including a compromised mucosal membrane barrier, bacterial infiltration, production of reactive oxygen species, and a potent local immune response [12]. The multiple beneficiary effects of melatonin on GI health [17,19,20,24,52] make it an interesting candidate drug (alone or in combination) to alleviate symptoms and effects associated with mucositis. The low price of melatonin and its high safety margin also enable this oral supplementary compound to have possibilities for broad use.

In conclusion, this rat study showed that melatonin receptor activation in the intestine mediated a reduction in surfactant-induced increases in intestinal mucosal permeability. It was also shown that nicotinic acetylcholine receptors in the enteric nervous system were involved in this process, by a mechanism using pathways not influenced by melatonin receptor activation.

## 4. Materials and Methods

### 4.1. Chemicals and Drugs

Ethanol, 5-ethyl-5-(1′-methyl-propyl)-2-thiobarbiturate (Inactin^®^), mecamylamine, melatonin, and SDS were purchased from Sigma-Aldrich (St. Louis, MO, USA). Luzindole was purchased from Tocris Bioscience (Bristol, UK). Sodium phosphate dibasic dihydrate (Na_2_HPO_4_∙2H_2_O), potassium dihydrogen phosphate (KH_2_PO_4_), sodium hydroxide (NaOH), and sodium chloride were purchased from Merck KGaA (Darmstadt, Germany). ^51^Cr-EDTA was purchased from PerkinElmer Life Sciences (Boston, MA, USA). Parecoxib (Dynastat^®^) was obtained from Apoteket AB (Uppsala, Sweden).

### 4.2. Study Formulations

An isotonic (290 mOsm) phosphate-buffered (pH 6.5, 8 mM) perfusate solution was prepared (perfusion solution) either with or without 5 mg/mL SDS (17.3 mM). To the perfusate solutions was added: (i) melatonin (100 µM), (ii) luzindole (100 µM), and/or (iii) mecamylamine (100 µM).

Ethanol stock solutions (65 mM) of melatonin, luzindole and mecamylamine were added to the perfusate solutions with final ethanol concentrations always below 0.5%. Osmolarity was determined by freezing-point decrement using a Micro Osmometer (Model 3MO; Advanced Instruments, Needham Heights, MA, USA).

Inactin was prepared at 500 mg/mL in deionized water. Parecoxib was prepared at 1 mg/mL in saline.

### 4.3. Animals, Anesthesia and Surgery

The study was approved by the local ethics committee for animal research (no. C64/16) in Uppsala, Sweden. Male Han Wistar rats (strain 273) from Charles River Co. (Germany), body weight 300–515 g were used. All animals were allowed to acclimatize for at least one week in the Animal Department prior to being used and were allowed water and food ad libitum. Housing conditions were 21–22 °C at a 12 h-12 h light-dark cycle. Surgical procedures and experimental setup of the rat SPIP experiment have been previously described [53]. In short, rats were anesthetized with an intraperitoneal injection of Inactin (180 mg/kg). To minimize preoperative stress, anesthesia was performed by experienced animal staff at the Animal Department, Biomedical Center, Uppsala, Sweden.

Body temperature was maintained at 37–38°C throughout experiments by a heating pad monitored by a rectal thermistor probe. An arterial catheter connected to a transducer-operated PowerLab system (AD Instruments, Hastings, UK) recorded systemic arterial blood pressure to control the general condition of the animals. An approximately 3 cm long abdominal incision was made and a jejunal segment (10–12 cm) was cannulated, placed outside of the abdomen [50] and covered with polyethylene wrap. At 30 min after surgery, parecoxib 10 mg/kg was given intravenously (iv) to reverse the surgery-induced paralysis of the intestine [54].

### 4.4. Perfusion Study

After completion of surgery, ^51^Cr-EDTA was administered iv as a bolus of 75µCi (0.4 mL), followed by a continuous iv infusion at a rate of 50 µCi per hour (1 mL/h) throughout the experiments. For the first 45 min following surgery, the jejunal segment was single-pass perfused with phosphate-buffered perfusate solution (pH 6.5, 8 mM, 37 °C) to allow for cardiovascular, respiratory, and intestinal functions to stabilize before experiments were commenced. The length and wet tissue weight of each intestinal segment were determined after the experiment. The luminal perfusion rate was at all times 0.2 mL/min (peristaltic pump, Gilson Minipuls 3, Le Bel, France).

**Figure 3:**
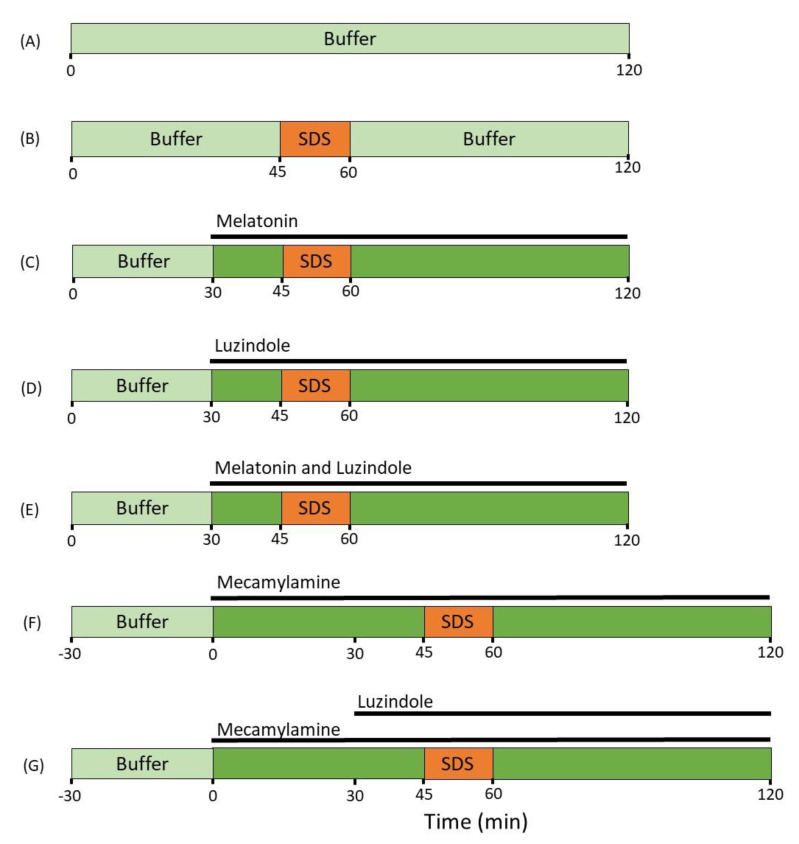
The luminal compositions, conditions, and treatments of the seven different experimental groups. The jejunal segment of rats (*n* = 6 in each group) was single-pass perfused with a pH 6.5 saline buffer solution without (**A**) or with (**B**–**G**) adding 5 mg/mL SDS between 45 and 60 min. Melatonin (**C**), luzindole (**D**) or a combination of the two (**E**), were added from 30 min onward to evaluate their effects on the SDS-induced increases in intestinal mucosal permeability. In the two mecamylamine groups (**F**,**G**), an additional 30 min of control period was added before the beginning of the experiment to evaluate its effects on basal permeability. Mecamylamine alone (**F**) or in a combination with luzindole (from 30 min onwards, (**G**)), was added from 0 min onward to evaluate their effects on the SDS-induced increases in intestinal mucosal permeability.

Following the 45 min stabilization period, rats (*n* = 6) were allocated to one of the seven different SPIP experimental groups (Figure 3). In the control group, only control buffer was perfused for 120 min. In the SDS control group, control buffer was perfused from 0–45 and from 60–120 min, while SDS was perfused from 45–60 min. In three SDS and luminal treatment groups, control buffer was perfused from 0–45 and from 60–120 min, while SDS was perfused from 45–60 min, and either melatonin, luzindole, or melatonin and luzindole were perfused from 30–120 min.

In two SDS and luminal treatment groups, control buffer with mecamylamine was perfused from 0–45 and from 60–120 min, while SDS was perfused from 45–60 min, In one of these groups, luzindole was added to the perfusate and perfused from 30–120 min. In the two mecamylamine groups, an additional 30 min of control period was added before the beginning of the experiment to ensure that it imposed no change in the baseline intestinal permeability value.

All experimental periods started with a rapid filling (<30 s) of the whole jejunal segment with the perfusate (37 °C, about 1.5 mL). The intestinal segment and perfusates were kept at 37 °C, and all outgoing perfusate was collected and weighed at 15 min intervals. Blood samples (<0.3 mL) were drawn from the femoral artery at the start (t = 0 min) and at the end (t = 120 min) of the perfusions. The blood samples were centrifuged (5000× *g*, 5 min) within 10 min, and the plasma and perfusates were analyzed for ^51^Cr activity.

### 4.5. Determination of Blood-to-Lumen Jejunal Mucosal ^51^Cr-EDTA Clearance

All luminal perfusates and blood plasma (at t = 0 and t = 120) were analyzed for ^51^Cr-EDTA activity (cpm) in a gamma counter (1282 Compugamma, CS, Pharmacia AB, Sweden). A linear regression analysis of the plasma samples was made to calculate a corresponding plasma value for each time point a perfusate sample was taken.

The blood-to-lumen CL_Cr-EDTA_ was calculated using Equation (1) [55]:

(1) $${\mathrm{CL}}_{\mathrm{Cr}-\mathrm{EDTA}}=\frac{C_{\mathrm{perfusate}}\;\times {\;Q}_{\mathrm{in}}}{C_{\mathrm{plasma}}\times \mathrm{tissue weight}}\;\times \;100$$

where C_perfusate_ and C_plasma_ are the activities (cpm/mL) in the perfusate and plasma, respectively, and Q_in_ is the flow rate (mL/min) into the segment. CL_Cr-EDTA_ is expressed as mL/min/100 g wet tissue weight. For the evaluation of CL_Cr-EDTA_ over time, CL_Cr-EDTA_ values were normalized against the average value of all groups during the 45 min control period. The area under the CL_Cr-EDTA_ over time curve between 45 and 120 min (total CL_Cr-EDTA_) was then calculated using non-compartmental analysis in GraphPad Prism version 8.4.0 for windows (La Jolla, CA, USA).

### 4.6. Statistical Analysis

Based on previous studies, a sample size of six rats was used in the CL_Cr-EDTA_ experiments. All descriptive statistics are presented as the mean ± standard error of the mean (SEM). The total CL_Cr-EDTA_ values of the different groups were compared using a Brown–Forsythe and Welch ANOVA with a Dunnett multiple comparison test. For the MABP data, a paired t-test was used to compare differences between the first (0–15 min) and last (105–120 min) measured time intervals within each group. Differences were considered statistically significant at a *p*-value < 0.05.

## Figures and Tables

**Table 1:** The mean (±SEM) arterial blood pressure (MABP) of the seven different groups (*n* = 6) during the rat single-pass perfusion experiments. The interval 0–120 min shows the average throughout the full experiment, 0–15 min during the first 15 min, and 105–120 during the last 15 min.

Groups	MABP (mmHg) (0–120 min)	MABP (mmHg) (0–15 min)	MABP (mmHg) (105–120 min)
Control	111 ± 5	113 ± 2	113 ± 7
SDS	100 ± 4	104 ± 4	97 ± 5
SDS and melatonin	104 ± 5	109 ± 8	99 ± 3
SDS and luzindole	100 ± 5	100 ± 6	105 ± 6
SDS, melatonin and luzindole	111 ± 8	112 ± 10	112 ± 9
SDS and mecamylamine	79 ± 6	95 ± 3	77 ± 7 *
SDS, mecamylamine and luzindole	93 ± 4	91 ± 7	93 ± 2

* *p* < 0.05 compared with the 0–15 min value in the same group (*t*-test).

## Data Availability

The data presented in this study are available on request from the corresponding author.
